# Use of magnetic resonance imaging combined with gene analysis for the diagnosis of fetal congenital heart disease

**DOI:** 10.1186/s12880-019-0314-8

**Published:** 2019-01-25

**Authors:** Lishun Wang, Hongyan Nie, Qichen Wang, Guoliang Zhang, Gang Li, Liwei Bai, Tianshu Hua, Shuzhang Wei

**Affiliations:** 1 Department of Radiology, Maternity and Child Care Center of Qinhuangdao, Qinhuangdao Maternal and Child Health Hospital, Qinhuangdao, Hebei 066000 People’s Republic of China; 2 Department of Ultrasound, Maternity and Child Care Center of Qinhuangdao, Qinhuangdao Maternal and Child Health Hospital, Qinhuangdao, Hebei 066000 People’s Republic of China; 3 Department of Anesthesiology, Maternity and Child Care Center of Qinhuangdao, Qinhuangdao Maternal and Child Health Hospital, qinhuangdao, Hebei 066000 People’s Republic of China; 4 Prenatal Diagnosis Center, Maternity and Child Care Center of Qinhuangdao, Qinhuangdao Maternal and Child Health Hospital, Qinhuangdao, Hebei 066000 People’s Republic of China; 5 Department of Epigenetics, Maternity and Child Care Center of Qinhuangdao, Qinhuangdao Maternal and Child Health Hospital, Qinhuangdao, Hebei 066000 People’s Republic of China; 6 Department of Radiology, Maternity and Child Care Center of Liuzhou, Liuzhou, Guangxi Zhuang Autonomous Region 545000 People’s Republic of China

**Keywords:** Fetus deformity, Congenital heart disease, Magnetic resonance imaging, Gene detection

## Abstract

**Background:**

Fetal deformity is a disease caused by abnormal chromosome structure, which may be influenced by genetic factors as well as the maternal and external environment. Magnetic resonance imaging (MRI) may be used to effectively diagnose fetus deformities. However it has been reported that gene analysis is a more accurate diagnostic method. The aim of the present study was to investigate the effectiveness of MRI in combination with gene analysis for the diagnosis of fetal congenital heart disease, a form of fetus deformity.

**Methods:**

MRI, array comparative genome hybridization analysis and fluorescence in situ hybridization were used to analyze the effectiveness of the two methods in a total of 78 pregnant women with suspected fetal congenital heart disease.

**Results:**

Our findings demonstrated that the combination of MRI and gene analysis resulted in significantly improved diagnostic accuracy, sensitivity and specificity for fetal congenital heart disease compared with either method alone. MRI combined with gene analysis confirmed 42 fetuses with pulmonary stenosis, 24 with aortic stenosis and 12 healthy fetuses, which was significantly improved compared with MRI or gene analysis alone. It was also observed that gene analysis was a more efficient method of diagnosis compared with MRI; however, the combination of the two methods was the most effective.

**Conclusion:**

In conclusion, the results of the present study suggest that MRI combined with gene analysis may be a more effective diagnostic method for fetal congenital heart disease compared with the current protocol.

## Background

Fetal deformities are one of the most common types of congenital malformation and may be caused by great vessel disorder or dysplasia formation [1, 2]. There are different types of fetal deformity, including Down’s syndrome, congenital heart disease, neural tube defects, cleft lip and palate, additional fingers or toes and hydrocephalus [3]. Previous studies have indicated that the underlying pathologies of fetal deformities are complex and may include genetic factors and external environment factors [4, 5]. Congenital heart disease may be divided into three types: No shunt (pulmonary and arterial stenosis), left to right shunt (ventricular septal defect, atrial septal defect and patent ductus arteriosus) and right to left shunt (tetralogy of fallot and transposition of great vessels) [6]. Congenital heart disease is the most common type of congenital malformation and accounts for ~ 28% of all congenital malformations [7, 8]. However, the diagnostic efficacy of congenital heart disease is often suboptimal, failing to enable early clinical intervention.

Magnetic resonance imaging (MRI) is the most common diagnostic method in the evaluation of congenital heart disease [9]. The safety and imaging quality of MRI has been confirmed in pediatric and adult patients with congenital heart disease and pacemakers [10]. Porayette et al. [11] reported that MRI effectively identified hemodynamic changes with acute maternal hyperoxygenation in human fetuses with and without congenital heart disease. In clinics, left ventricular eccentricity index measured by cardiac MRI has been used to assess right ventricular hemodynamics and myocardial fibrosis in congenital heart disease, suggesting that MRI is a more effective diagnostic method for congenital heart disease compared to computed tomography (CT) and ultrasonography [12]. However, MRI alone is insufficient to confirm congenital heart disease in a fetus [13].

Gene analysis may be used to detect > 150 types of human disease, including cardiovascular system, cancer and other rare diseases (epilepsy and phenylketonuria) [14–16]. Gene analysis may be used for prenatal diagnosis for congenital heart disease via the molecular analysis of a splicing mutation (c.2639 + 1G > C) in the splice donor site of intron 22 of the tuberin (TSC2) gene in the mother and the fetus [17]. Chen et al. [18] reported that MRI was used to prenatally diagnose rhabdomyomas and cerebral tuberous sclerosis in one fetus of a dizygotic twin pregnancy, which was associated with a frameshift mutation in the TSC2 gene. However, to the best of our knowledge, no previous studies have investigated the role of the c.2639 + 1G > C splice site mutation in intron 22 of the TSC2 gene in the diagnosis of congenital heart disease.

In the present study, the diagnostic efficacy of MRI used in combination with gene analysis for fetal congenital heart disease was investigated. Previous studies have indicated that MRI may be used to detect heart defects in fetuses from a mean gestational age of 37 weeks [19–21]. The presence of a heart defect may be a pathogenic factor that affects brain development in the fetus due to a reduction in cerebral oxygenation [22]. The present study revealed that MRI combined with gene analysis improved diagnostic accuracy in fetuses with congenital heart disease compared with either method alone. The importance of MRI and gene analysis in the diagnosis of fetal congenital heart disease was highlighted, which may provide a clinical foundation to evaluate the risk of maternal manifestations in fetuses with congenital heart disease.

## Methods

### Study participants

A total of 78 pregnant women with suspected fetal congenital heart disease (age, 32.4 ± 8.4) and 78 healthy pregnant volunteers (age, 30.6 ± 9.5) were recruited for the present study at the Maternity and Child Care Center of Liuzhou (Liuzhou, China) between May 2012 and June 2015. The present study was approved by the Ethics Committee of the Maternity and Child Care Center of Liuzhou. Patients with a history of family heart disease were excluded from the study. All patients and healthy volunteers provided written informed consent prior to their inclusion within the study. Individuals with diabetes mellitus, nephritis, human immunodeficiency virus infection and chronic renal failure were excluded from the study.

### Clinical description

Blood samples (10 ml) from mother and amniotic fluid (5 ml) were collected from all study participants on week 40 of gestation as previously described [23]. Genomic DNA was extracted from blood leucocytes and all fetuses as described previously [24]. DNA was extracted from blood leucocytes using a DNA Extract All Reagents Kit (Applied Biosystems™) according to the manufacturer’s protocol (Qiagen China Co., Ltd., Shanghai, China).

### Fetal MRI

Fetal MRI was performed at gestational week 40 using a 1.5-Tesla Siemens Essenza scanner (Siemens, China) with a 8-channel phased-array coil. Subjects in supine position were guided to breathe smoothly. T2-Trufi and T2-Haste sequences were used to acquire axial, coronal and sagittal images of the fetus heart. The following parameters were used: T2-Trufi: repetition time (TR) = 3.6–4.2 ms, echo time (TE) = 1.0–1.8 ms, matrix size = 512 × 512; T2-Haste: TR = 1150-1450 ms, TE = 42-145 ms, matrix size = 512 × 512. Slice thickness = 4-6 mm, inter-slice gap = 0–0.5 mm. The duration of MRI acquisitions used in this study was 0.5–2 s per image. All methods and experiments were performed in accordance with relevant guidelines and regulations. The study was approved by the Institutional Review Board and the Committee on Clinical Investigation and written informed consent was obtained from all participants. Images was processed using Siemens Syngo D14 software. Diagnosis of congenial heart diseases was made by an experienced radiologist based on T2-hypotensities in the left ventricular free wall and left atrium.

### Array comparative genome hybridization (aCGH) analysis

Whole-genome aCGH analysis was performed on the DNA extracted from the fetuses using NimbleGen ISCA and cytogenetic array (Roche Sequencing, Pleasanton, CA, USA). This kit includes 630,000 probes and a median resolution of 1.5 × 10^20^ kb across the entire genome. The kit was used according to the manufacturer’s protocol. All data were presented using the Nexus 6.1 (BioDiscovery, Inc., El Segundo, CA, USA).

### Fluorescence in situ hybridization (FISH)

Metaphase FISH analysis was performed on cultured amniocytes derived from the amniotic fluid and maternal blood for 12 h at 37 °C, using a 17q12-specific bacterial artificial chromosome (BAC) probe RP11–143E18 (dye, Texas red, for 1 h at 37 °C) (gene location: 35,985,121e36,129,469) and a control 17q25.3-specific BAC probe RP11-388C12 (dye, FITC green) (80,606,711e80,718,184) (hg19) according to the standard FISH protocol [25].

### Statistical analysis

All data are presented as the mean ± standard deviation of three independent trials for each experiment. All data were analyzed using SPSS Statistics software version 19.0 (IBM Corp., Armonk, NY, USA). A receiver operator characteristic curve was generated to determine the cut-off point for optimal sensitivity and specificity. Statistical differences between groups were assessed by one-way analysis of variance with a post-hoc Dunnett’s test. *P* < 0.05 was considered to indicate a statistically significant difference.

## Results

### Characteristics of the study participants

A total of 156 pregnant women were recruited for the present study; 78 were healthy volunteers and 78 had suspected fetal congenital heart disease. No statistically significant differences were observed in the blood pressure, blood glucose and heart rate between the suspected fetal congenital heart disease group and the healthy volunteers (Table 1). The characteristics of the study participants are summarized in Table 1.

**Table 1:** Characteristics of pregnant women with suspected fetal congenital heart disease and healthy volunteers

Characteristic	Group		*P*-value
	Patients (*n* = 78)	Healthy (*n* = 78)	
Age (years)	32.4 ± 8.4	30.6 ± 9.5	0.58
Pregnancy duration (weeks)	40	40	1.00
Blood pressure (mmHg)	115.2 ± 10.6	113.8 ± 12.2	0.68
Blood glucose (mmol/l)	8.4 ± 2.8	8.0 ± 1.7	0.72
Heart rate (beats/mim)	132 ± 12	128 ± 13	0.75

Data are presented as the mean ± standard deviation

### Diagnosis of suspected fetal congenital heart disease using MRI

**Fig. 1:**
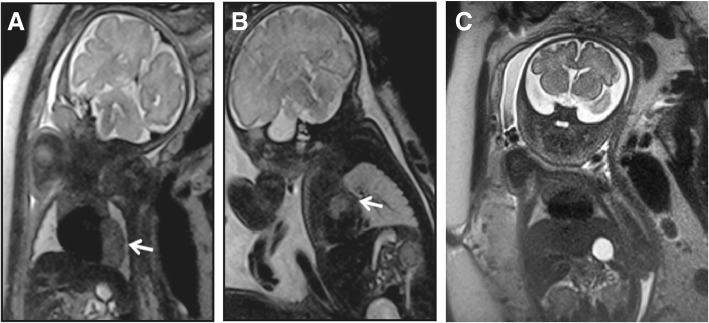
Use of MRI for the diagnosis of suspected fetal congenital heart disease in pregnant women. Fetal MRI revealed (**a**) T2-hypointense lesions on the left ventricular free wall and (**b**) lesions on the left atrium in a case of suspected fetal congenital heart disease. Lesions were indicated by white arrows. (**c**) Fetal MRI of a normal fetus as a control. All images were acquired using T2-Haste sequence using the following parameters: TR = 1000 ms, TE = 92 ms, slice thickness = 4 mm

The diagnostic efficacy of MRI for fetal congenital heart disease was investigated. Fetal MRIs were performed at gestational week 40. The fetal MRIs revealed T2-hypointense lesions arising from the left ventricular free wall (Fig. 1a) and left atrium (Fig. 1b) in all women with suspected fetal congenital heart disease. The fetal MRI images of healthy subjects are shown in Fig. 1c, in which hypointense lesions were absent. These results indicate that MRI was able to effectively diagnose fetal congenital heart disease.

### Diagnosis of suspected fetal congenital heart disease using gene analysis

**Fig. 2:**
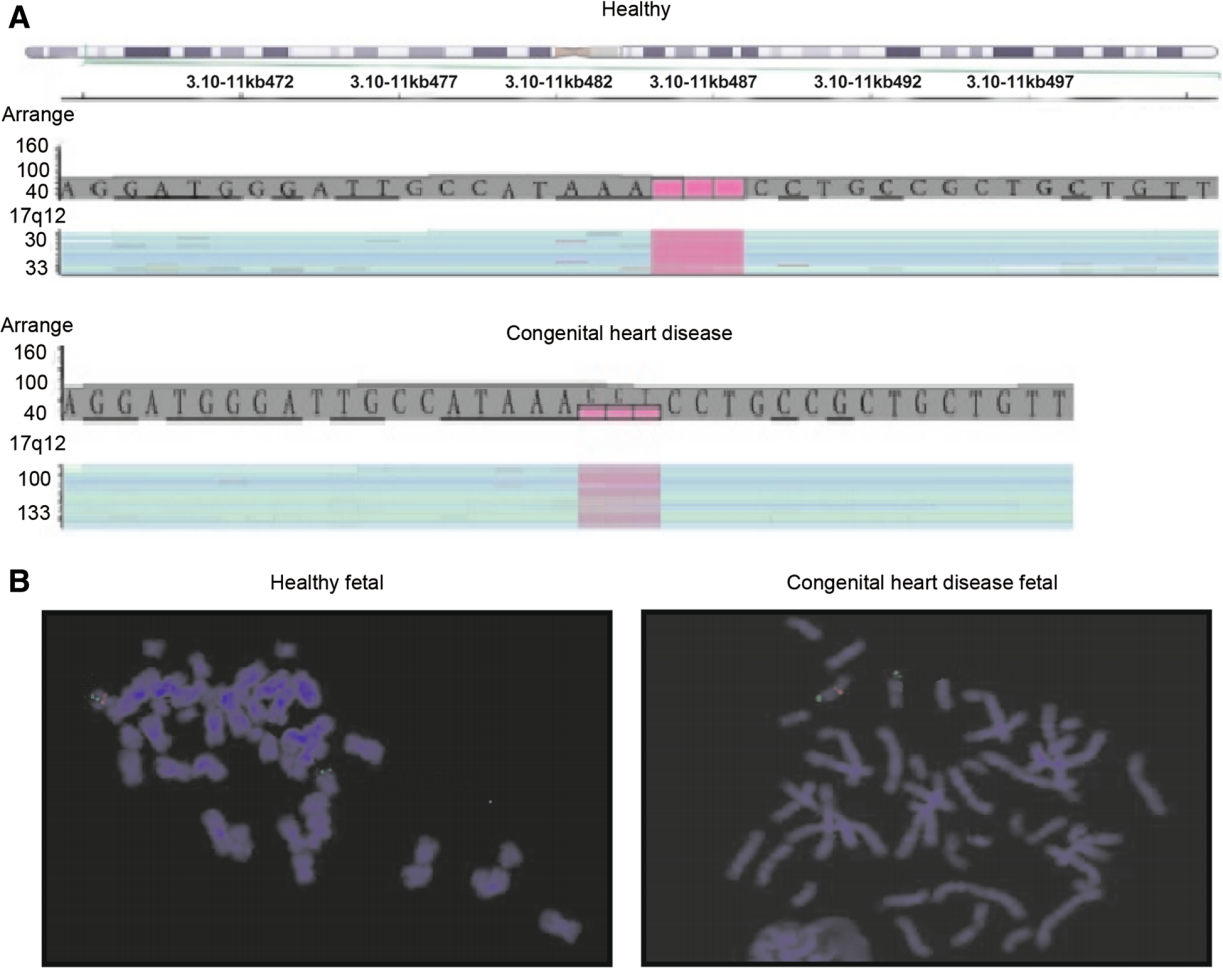
Use of gene analysis for the diagnosis of suspected fetal congenital heart disease in pregnant women. (**a**) Detailed view of the region with A-OH (AOH) in 3p:arr (hg19) 3p26.1p25.3p25.2 (6206901–12,352,468) × 2 hmz. (**b**) DNA sequences for exon 23 of the *FANCD2* gene of the fetus and mother, visualized with Golden Helix GenomeBrowse 2.0.2 software

All patients were referred for genetic counseling and genetic studies were conducted to determine the diagnosis of the fetus. A 1.75-Mb deletion at 17q12, including haploinsufficiency of LIM/homeobox protein Lhx1 and hepatocyte nuclear factor 1-β, was investigated using aCGH analysis. The whole-genome analysis revealed a 1.54-Mb deletion at 17q12, or arr (hg19) 17q12 (gene location: 34,814,526e36,355,604) in fetal congenital heart disease (Fig. 2a). Differences in 17q12 signaling were observed in fetal blood lymphocytes from the suspected congenital heart disease group and the healthy group using a 17q12-specific BAC probe. Congenital heart disease fetal showed lower expression of 17q12 than healthy control (Fig. 2b). These results indicated that gene analysis was effective in diagnosing fetal congenital heart disease.

### Diagnostic efficacy of MRI in combination with gene analysis for the diagnosis of suspected fetal congenital heart disease

**Fig. 3:**
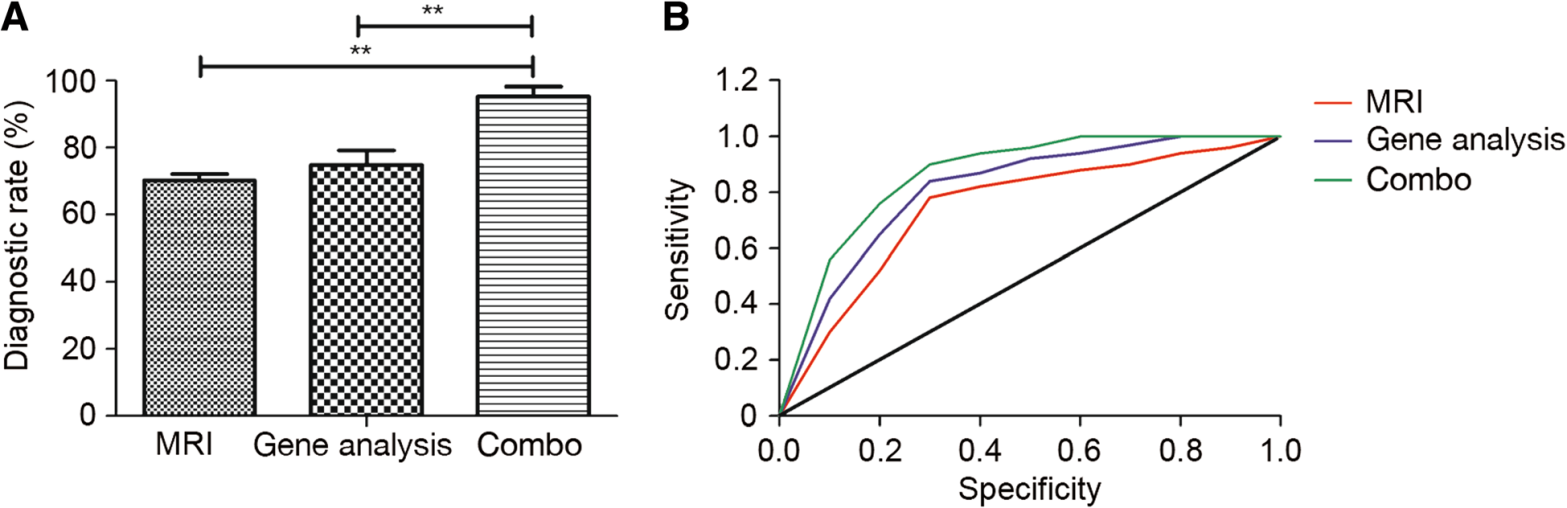
Analysis of the efficacy of MRI with gene analysis for the diagnosis of patients with suspected fetal congenital heart disease. (**a**) Using a combination of MRI and gene analysis resulted in a significantly higher confirmed diagnostic rate compared with MRI or gene analysis alone. (**b**) MRI combined with gene analysis increased the diagnostic sensitivity and specificity for fetal congenital heart disease compared with MRI or gene analysis alone. Black line indicates the diagonal reference line. ^**^P < 0.01

The confirmed diagnostic efficacy of combined MRI and gene analysis for pregnant women with suspected fetal congenital heart disease was evaluated. The combination of MRI and gene analysis had a significantly higher confirmed diagnostic rate compared with MRI or gene analysis alone for patients with suspected fetal congenital heart disease (*P* < 0.01; Fig. 3a). It was revealed that MRI used in combination with gene analysis increases the sensitivity and specificity of diagnosis for fetal congenital heart disease compared with MRI or gene analysis alone (Fig. 3b).

In the present study, MRI used in combination with gene analysis successfully diagnosed 42 fetuses with pulmonary stenosis, 24 with arterial stenosis and 12 healthy fetuses, which is a significant improvement compared with MRI or gene analysis alone (Table 2). These results indicate that the combination of MRI and gene analysis presents a more accurate, sensitive and specific diagnostic method for pregnant women with suspected fetal congenital heart disease compared with the use of MRI or gene analysis alone.

**Table 2:** Diagnostic efficacy of MRI and gene analysis for pregnant women with suspected fetal congenital heart disease

Diagnosis	No. positive diagnoses using different methods		
	MRI	Gene analysis	Combination
Pulmonary stenosis	32	37	42^a,b^
Arterial stenosis	15	18	24^a,b^
Healthy	31	23	12

*MRI* magnetic resonance imaging. ^a^*P* < 0.05, ^b^*P* < 0.01

## Discussion

Congenital heart disease is one of the most common types of fetal deformity and may be caused by a variety of pathological factors, including a change in chromosomal structure or a gene mutation [26, 27]. Diagnosing congenital heart disease is an important part of prenatal screening [28, 29]. In the present study, the diagnostic efficacy of combined MRI and gene analysis was investigated in pregnant women with suspected fetal congenital heart disease. The results demonstrated that the combined use of MRI and gene analysis resulted in a significantly higher diagnostic efficacy for fetal congenital heart disease compared with single diagnosis using MRI or gene analysis alone. The results also suggest that a 1.54-Mb deletion at 17q12 in fetal genome may be regarded as a potential diagnostic gene marker for fetal congenital heart disease.

At present, MRI is widely used to diagnose human diseases and has become more effective than CT and X-rays for the early diagnosis of human tumors, heart diseases and cerebrovascular diseases [11]. Cheng et al. [30] reported that comprehensive motion-compensated highly accelerated 4D flow MRI with ferumoxytol enhancement may be used to improve diagnoses of pediatric congenital heart disease. Rose et al. [31] used a 4D flow MRI to reveal significant changes in the cardiovascular hemodynamics of complex congenital heart disease. The results of the present study confirm that MRI is an effective method for diagnosing fetal congenital heart disease in pregnant women, as it revealed T2-hypointense lesions arising from the left ventricular free wall and left atrium of the fetuses.

Gene analysis is regarded to be an effective method for the diagnosis of patients with genetic disorders [32–34]. Molecular diagnosis of congenital heart disease has focused on loss-of-function mutations, which may be used to identify the causative genes of congenital heart disease and therefore personalize the treatment administered [35]. Chen et al [36] demonstrated the prenatal diagnosis and molecular genetic analysis of short rib-polydactyly syndrome type III (Verma-Naumoff) for short limbs in the fetus by detection of a mutation in the *NEK1* gene. In the present study, the diagnostic efficacy of gene analysis for fetal congenital heart disease was confirmed. A previous study reported that the detection of recurrent transmission of 17q12 microdeletion by aCGH may be used to analyze hydronephrosis, hydroureter and multicystic kidney and variable clinical spectra in families [37]. In the present study, gene analysis revealed that 1.54-Mb deletion at 17q12, or arr (hg19) 17q12 (34,814,526e36,355,604) was observed in fetus in pregnant women with suspected fetal congenital heart disease. The present study observed differences in the 17q12 signal using a 17q12-specific BAC probe in cultured amniocytes and maternal blood lymphocytes, which suggests that gene analysis is more efficient for the diagnosis of suspected fetal congenital heart disease (Table 2).

## Conclusion

In conclusion, the present study indicates that MRI combined with gene analysis is a more accurate, sensitive and specific diagnostic method for fetal congenital heart disease compared with MRI or gene analysis alone. In addition, the molecular cytogenetic characterization of a recurrent 17q12 microdeletion in fetal congenital heart disease was reported, which may serve as a potential diagnostic indicator. However, further studies should be performed in a larger sample population to identify the diagnostic efficacy of combination of MRI and gene analysis.

## Abbreviations

aCGH: Array comparative genome hybridization
BAC: Bacterial artificial chromosome
CT: Computed tomography
FISH: Fluorescence in situ hybridization
MRI: Magnetic resonance imaging
